# Supplementary material for: Genetic comparisons of interleukin-17 reveal a framework for complex signaling evolution
Source: bioRxiv. 2026 Apr 14:2026.04.13.718218. Preprint. [Version 1] doi: 10.64898/2026.04.13.718218 (PMC13105070; doi:10.64898/2026.04.13.718218)
Supplement: Supplement 3 [file media-3.zip › Supplemental files for paper/ERC/IL17xIL17_comparisons/IL17RD_IL17RD.pdf]

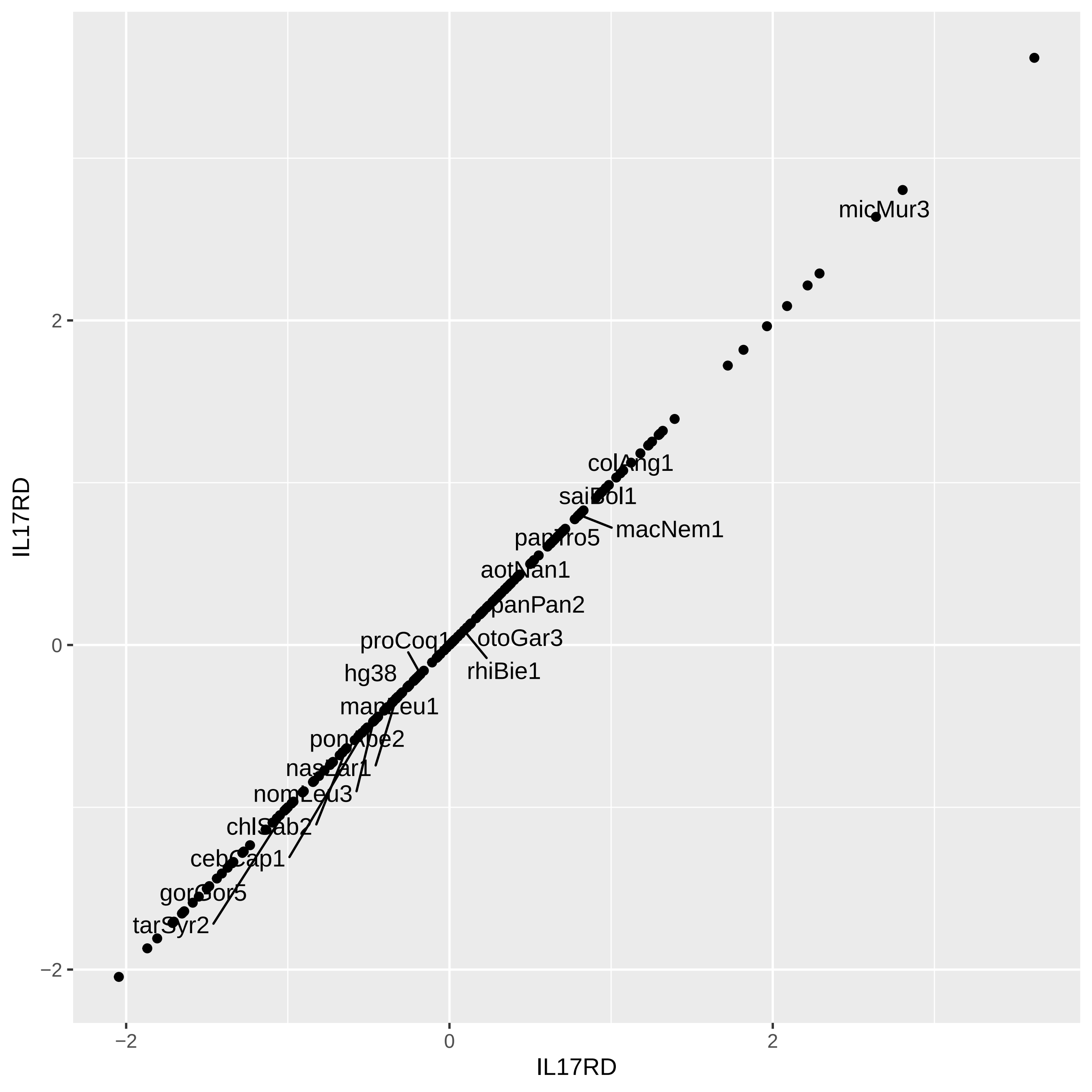

micMur3
colAng1
saiBol1
macNem1
panTro5
aotNan1
panPan2
otoGar3
proCoq1
rhiBie1
hg38
manLeu1
ponAbe2
nasLar1
nomLeu3
chlSab2
cebCap1
gorGor5
tarSyr2
IL17RD
IL17RD
2
0
−2
−2
0
2
